# A Lecture on Menorrhagia, Vel Hæmorrhagia Uteri, or Uterine Hæmorrhage

**Published:** 1839-04-06

**Authors:** N. Chapman

**Affiliations:** Professor of the Theory and Practice of Physic, in the University of Pennsylvania


### MEDICAL EXAMINER.

DEVOTED TO MEDICINE, SURGERY, AND THE COLLATERAL SCIENCES.

No. 14.] PHILADELPHIA, SATURDAY, APRIL 6, 1839. [Vol. II.

### A LECTURE ON MENORRHAGIA, VEL HÆMORRHAGIA UTERI, OR UTERINE HÆMORRHAGE. *By* N. Chapman, M. D., *Professor of the Theory and Practice of Physic, in the University of Pennsylvania.*

(Concluded from page 199.)

Connected as this hæmorrhage is, with an active, and even, perhaps, inflammatory condition, it is often circumstanced differently. We have, in the latter cases, proofs of a debilitated, and sometimes a vitiated state of system. The pulse is feeble and quick, the respiration hurried on the slightest exertion, the skin damp and cold, pallid or sallow, and doughy, with, in protracted instances, œdema of the lower extremities, and particularly of the feet, in the evening. That, however, which distresses most, is a constant pain in the lumbar region, sometimes acute, though more frequently dull, with a sense of weakness in the back, which may be so great as even to prevent the erect position. Effusions are occasionally very profuse, the blood thin or watery, and inasmuch, from the existing debility, any further expenditure of it must be detrimental, it is to be checked as speedily as possible.

General bleeding here, can rarely be practised. But in those instances, where, evidently, there is much local congestion, though the system may be weak, cupping over the lumbar region is allowable,—and, at all events, dry cupping, or a blister, or both, in the same position, may be applied. Excepting the nitrate of potash, all the other medicines in the active, are adapted to this hæmorrhage, and especially the topical means which were enumerated. There are, moreover, some other articles given internally, and among these is alum. That it has been found useful, it is hard to doubt. The earliest appropriation of it, indeed, was to uterine hæmorrhage, by Van Helmont, who acquired immense fame by the cures effected by it. The dose is from three to five grains, with a portion of opium, to be repeated according to the emergency. Though it is usual to combine it with kino or catechu, no advantage is gained, and it were better to give these articles separately. They are not, however, in any mode of administration, highly appreciated by me. The extract or tincture of rhatany has, perhaps, stronger claims to attention, and the elixir of vitriol is undoubtedly sometimes serviceable. Neither of the gallic acid, nor cresote, lately so strongly praised, have I any personal experience in this application of them.

It is now proper that I should deliver some account of the use of emetics, and particularly, as the practice is not without claims to originality. This inactive hæmorrhage may be alarming in its immediate tendency,—it is always seriously injurious to health, and often proves intractable to the customary mode of treatment. Embarrassed by a case of the kind, which had resisted the best efforts of some other practitioners, I determined to venture on the experiment with emetics. To this conclusion I was led by the reflection, that there is no peculiarity in uterine hæmorrhage not reconcileable to the common principle on which I had conducted the cure of other forms of the disease. It struck me, that by the revulsion of vomiting, distinct from the secondary effects of the process, the flow of blood might be checked, and that in the interval of its recurrences, by occasional repetitions of the remedy, the uterus reinstated in its secretory functions. Emetics, I was also aware, are among the most active and certain of the emmenagogues, by which I mean, an immediate power to arouse the energies, or otherwise to reinvest the uterus with the faculty of secretion, when suspended or perverted. Having seen their salutary agency in this respect, as well in amenorrhœa, as fluor albus, I indulged the hope, that if in these cases they can revive a natural action, or rectify a depraved one, so they might be serviceable in the same way in hæmorrhage.

The case to which I have referred, occurred at the close of 1827. It was that of a lady in the prime of life, from a distant part of the country, who came to consult me. Her appearance was sickly, and she told me, that from her marriage, a year and a half before, she had been subject to hæmorrhage, at first, inconsiderable and monthly, progressively, however, increasing in quantity, and renewed at shorter intervals, till it had become so copious, on some occasions, as to endanger her existence. This distressing situation was greatly aggravated by her sterility.

The ordinary routine of remedies having been ineffectually exhausted, I suggested a trial of a course of treatment in conformity to the views just presented. With this advice she returned home, promising strictly to adhere to it. Two months afterwards I received a letter from her, in which she informed me, that on her journey, she had a comparatively slight hæmorrhage,—though, under an apprehension of its increasing, recourse was had to an emetic, which promptly suppressed it;—that, by this favourable result, fresh confidence was inspired in the proposed practice, and she had accordingly taken six emetics at the interval of eight days each, when regular menstruation returning, her general health was sensibly improved. Encouraged by this communication, I have since pursued the practice to some extent, and though not uniformly successful, it has proved sufficiently so, to claim, in my opinion, great respect.

To excite vomiting I have uniformly employed ipecacuanha, and, perhaps, no other article is so well adapted to the case. It affords me pleasure to find, from a late publication, that the emetic practice, as well as the preference of this article, is fully sanctioned by two very eminent practitioners of Europe. M. Coffin, of Paris, declares this, as the result of ample experience,—and which is abundantly confirmed by Dr. Osborne, of Dublin, who states that a scruple of ipecacuanha, taken in the evening, and followed by an acidulated saline purgative in the morning, checked the discharge very speedily, and when it returned, the emetic repeated once or twice, never failed to complete the cure. Tartarized antimony had no such effect. As to the applicability of the practice, he perceived no difference, whether the hæmorrhage was decidedly active, or the reverse, it being alike successful.

Checked in any manner, the next consideration is to prevent the recurrence of the hæmorrhage, or, in other words, to effect a thorough cure. Before commencing the treatment, the pathological condition on which this disposition to effusion depends, must be ascertained. Either of a phlogistic, or active congestive nature, the loss of blood, generally or locally, from time to time, with the other means of reduction, including low diet, are, perhaps, only demanded. But in an opposite state, or one of enervation or relaxation, an essentially different course becomes proper.

Of the utility of emetics, as well to arrest the flow, as to obviate its recurrence, I have already spoken. Cathartics which act on the lower portion of the bowels, and indirectly on the uterus, have also been employed. The aloetic preparations are of this description, and among the very best of them are the hiera picra and elixir proprietatis. These articles are designed to operate not so much as evacuants, as by an impression on the vessels of the uterus, supposed to be promotive of the menstrual function. Governed by the same principle, emmenagogues of a more decisive character have been directed. But whatever may be the merit of these, of which I am exceedingly distrustful, the tonics are less equivocal, and more commonly employed. Great reliance was once placed on the Peruvian bark, and its ordinary preparations, now superseded by the sulphate of quinine.

But, above all, should our trust be reposed in the chalybeates. Excellent in every variety of this enfeebled state of the system, it is when exsanguineous, or cachectic, that they are best adapted. The phosphate of iron is the most valuable alone, or with the quinine, though the muriated tincture, the subcarbonate, the sulphate, the tartrate, the prussiate, and hydriodate, are all in repute. To aid this plan of combination, nutritious, though not a heating or stimulating diet, may be suggested, with the use of the cold bath, moderate exercise, and whatever else is calculated to renew or improve health.

Not, however, succeeding, some radical derangement of the uterus is to be suspected, and without much investigation of the nature of the lesion, mercury has been directed with a view to its reparation. Cautiously administered, there are states in which it might be servicable, as those in which it is beneficially resorted to in other secretory organs. Even under the supposition of such uterine depravation, a general or indiscriminate application of it could not, however, fail of producing infinite mischief. With these very states, there is, in many instances, a pervading bad habit of body, both of the solids and blood, the latter, especially, being thin and impoverished, by the loss of its fibrin and globules, and here mercury would be the most pernicious of articles. Employed, at all, it must be reserved for cases where the integrity of the constitution has not materially suffered.

In the management of this hæmorrhage, the principal design should be to replace the system in a healthy state, on the accomplishment of which, menstruation usually returns, and with it, the hæmorrhagic tendency ceases. Never ought it be forgotten, that the latter, existing during the period of life, when the former should be performed, it is seldom completely overcome, except by the restoration of the natural function.

Nor as a prophylactic of the outmst importance, must it escape notice, that on the hæmorrhage observing the law of periodicity with any degree of exactness, reverting, for instance, monthly, or at any stated interval, it may often be averted, by enjoining for a few days, in anticipation of an attack, a state of entire rest,—and where local uneasiness, or other signs of a congestive or phlogistic state prevails, by the loss of blood, generally or locally, a reduction of diet, and, perhaps, occasionally, an opiate. Examples, indeed, occur, in which the tendency to effusion is so continued, that the recumbent position is absolutely required for weeks or months together, to prevent its reappearance.

It remains to make a few remarks on a peculiar state of the disease. An irregularity in the discharges of the uterus, may be expected to a greater or less extent at the season of the cessation of the menses. The secretory office of this organ being about to terminate, it is imperfectly performed, and, consequently, we have some anormal secretion, or oftener pure blood, in the place of the catamenia, or a mixture of these fluids, and which is thrown out with no uniformity as to time or quantity. It happens, too, at this, or a later period, that we meet with cases where there is a small, though nearly constant oozing of blood, quaintly denominated by the late Professor Rush "a hemoptoe of the uterus." It may be suspected under such circumstances, particularly the latter, that there is something wrong in the condition of the womb, either chronic congestion, or phlogosis, and which, being neglected, leads to the formation of some more formidable lesion.

Cases of this nature are to be usually recognised by a sense of heat, and pain in the uterus, the latter extending to the lumbar region and lower extremities,—by the smallness of the discharge,—and in a more advanced stage, by an aggravation of the preceding symptoms, with depraved and offensive discharges. Doubts, however, existing, these may be removed by an examination *per vaginum*, when, in the commencement, the os tincæ, and, perhaps, the neck of the womb, will usually be found thickened, betraying increased sensibility to the touch, and subsequently, still more disease. Desirous of absolute certainty as to the nature and degree of the affection, this is attainable by the introduction of a *speculum*, lately contrived for the purpose, into the vagina, which brings the parts distinctly into view.

The mere suppression of the sanguineous discharge in the state just noticed, is, however, a subordinate consideration. Not excessive, it is even salutary, and must not be checked. The great object is to arrest the progress, or entirely relieve that condition from which it emanates. To this end, the most approved means are general and topical bleedings. The latter are usually made from the groins and vulva, though it is now sometimes effected by leeches to the uterus itself, as more effectual, applied by means of the speculum mentioned, which, however, I think questionable. As well on the general principle, that it is preferable to draw blood from the vicinity, than directly from the affected part, I have actually seen instances of uterine irritation by the bites of the leeches. Further, the treatment consists in an alterative use of mercury, low diet, principally of milk, with an avoidance of all exasperating causes.

In a later stage, when scirrhus and other serious organic degenerations have taken place, our chief reliance has been on arsenic, and the free exhibition of the narcotics, hemlock, stramonium, henbane, opium, &c. Little is to be anticipated from these remedies,—and such cases are now usually resigned to the resources of surgery, from which, however, I apprehend, scarcely more is derived. Notwithstanding all the vauntings of Lisfranc, and others, of the extraordinary success of their operations on the uterus, for the removal of diseased portions of it, great reason exists to distrust the integrity of these statements. As regards those of Lisfranc especially, there has been lately published, the solemn declaration of the resident surgeon of the hospital in which they occurred, that nearly the whole of the cases reported as cured, ended fatally. We have here a very striking illustration of the common and highly censurable conduct of certain surgeons, who, to acquire the *eclat* of a daring operation, proclaim, at once, the performance of it, and conceal the ultimate result, however ineffectual or disastrous it may be. It is a custom, to say the least of it, far "more honourable in the breach, than the observance," and from which, every one who is sensible of what is due to himself, or of the obligations to the profession, will turn with loathing and disgust.

